# Colonized *Aedes albopictus* and its sexual performance in the wild: implications for SIT technology and containment

**DOI:** 10.1186/1756-3305-6-206

**Published:** 2013-07-15

**Authors:** Dieng Hamady, Norrafiza Binti Ruslan, Abu Hassan Ahmad, Che Salmah Md Rawi, Hamdan Ahmad, Tomomitsu Satho, Fumio Miake, Wan Fatma Zuharah, Yuki FuKumitsu, Ahmad Ramli Saad, Sudha Rajasaygar, Ronald Enrique Morales Vargas, Abdul Hafiz Ab Majid, Nik Fadzly, Idris Abd Ghani, Sazaly AbuBakar

**Affiliations:** 1 School of Biological Sciences, Universiti Sains Malaysia, Penang, Malaysia; 2 Faculty of Pharmaceutical Sciences, Fukuoka University, Fukuoka, Japan; 3 Department of Medical Entomology, Faculty of Tropical, Medicine, Mahidol University, Bangkok, Thailand; 4 Faculty Science and Technology, Universiti Kebangsaan Malaysia, Bangui, Malaysia; 5 Department of Medical Microbiology, University of Malaya, Kuala Lumpur, Malaysia

**Keywords:** *Aedes albopictus*, Laboratory strain, Wild strain, Mating affinity, Containment

## Abstract

**Background:**

Mating is a physiological process of crucial importance underlying the size and maintenance of mosquito populations. In sterile and incompatible insect technologies (SIT and IIT), mating is essential for mass production, persistence, and success of released individuals, and is a central parameter for judging the effectiveness of SIT/IIT programs. Some mosquitoes have an enormous reproductive potential for both themselves and pathogens and mating may contribute to persistence of infection in nature. As *Aedes albopictus* can transmit flaviviruses both sexually and horizontally, and as infected insects are usually derived from laboratory colonies, we investigated the implications of mating between a long-term laboratory colony of *Ae. albopictus* and wild populations.

**Methods:**

Through a series of mating experiments, we examined the reproductive outcomes of sexual cross-affinity between laboratory-raised and wild adults of *Ae. albopictus*.

**Results:**

The results indicated appreciable mating compatibility between laboratory-reared and wild adults, and equivalent levels of egg production among reciprocal crosses. We also observed comparable larval eclosion in lab females mated with wild males, and increased adult longevity in female offspring from wild females|×|laboratory males crosses.

**Conclusions:**

Taken together, these data suggest that *Ae. albopictus* can preserve its reproductive fitness over a long period of time in the laboratory environment and has valuable attributes for SIT application. These observations together with the ability to successfully inseminate heterospecific females indicate the potential of *Ae. albopictus* to act as an ecological barrier if non-sterilized males are massively released in areas occupied by *Aedes aegypti*. The observed substantial reproductive fitness combined with the capability to reproduce both, itself and viruses illustrates the potential of *Ae. albopictus* to pose a serious threat if infected and released accidentally.

## Background

*Aedes albopictus* has spread worldwide [1,2] and its establishment in an area has often been associated with a decline, sometimes leading to local extinction, of the indigenous *Aedes* populations [1]. This mosquito transmits several arboviruses, including those responsible for yellow fever and various types of encephalitis. It is also a competent laboratory vector of more than 20 arboviruses [3,4], including Chikungunya virus [5,6]. However, this mosquito is best known as a vector for dengue viruses [7,8]. Dengue causes more human morbidity and kills more people than any other mosquito-borne virus globally [9,10]. The World Health Organization has estimated that more than 2.5 billion people are at risk of dengue infection [11] and recently classified this disease as a pandemic threat [12].

Many insect vectors, including *Ae. albopictus*, are maintained in laboratories for experimentation [13]. Although there are protocols for safe containment [14,15], there have been reports of escape of insect into the wild and subsequent public concern [13]. In addressing this issue, the World Health Organization has argued for the need to take eggs into account in containment strategies because they can transmit pathogens [16]. This is true for *Ae. albopictus* from which dengue serotype 2 virus has been isolated from field-collected males and female adults [17], indicting that the virus was transmitted from infected females to progeny via infection of the ovaries and eggs. The main strategy to stop the spread of such infections in human populations involves insecticide use [11]. However, the development of resistance has severely impeded the success of such strategies [18,19]. As the spectrum of effective insecticides has been drastically reduced and in the absence of effective vaccines, specific therapeutic treatments, and cures [20], the development of novel strategies to complement existing control measures has, therefore, become imperative. In particular, the control of *Ae. albopictus* with conventional methods appears particularly difficult due to the increased number of habitats in which it can thrive [21].

One approach being pursued is the sterile insect technique (SIT), which has been used to successfully control a number of insect pests [22,23]. SIT offers a promising strategy for dengue vector control [24], and much of this optimism is based on the results of a previous study [25]. These authors examined the use of SIT against *Ae. albopictus* in Italy. The results of pilot release of radiosterilized males in selected villages indicated a reduction of 72% in the wild population size. Another promising strategy, the incompatible insect technique (IIT), is being considered as an additional powerful tool to control populations of this species [21]. In both SIT and IIT, the persistence and success of released insects rely critically on successful sexual interactions with their wild counterparts. One problem associated with these technologies is the mating competitiveness of the released insects [19,26]. Thus, the ability to produce laboratory insects with high sexual efficiency in the wild remains a major challenge in SIT and IIT strategies. Therefore, better knowledge regarding the mating affi nity of laboratory-reared mosquitoes and their wild counterparts is required.

The SIT and IIT techniques are based on the release of sterilized laboratory-adapted individuals into the wild, an environment that is distinct from the laboratory environment [26,27]. The individuals are derived from colonies that have been maintained under laboratory conditions for long periods [19]. As colonization can cause abnormal mating behaviors, reduced genetic variation and fitness, sexual isolation, and genetic divergence between colonized and wild populations [28,29] as well as population bottlenecks [30], long-term colony maintenance may affect physiological and reproductive fitness. Despite a previous study regarding the sexual performance of male *Ae. albopictus*[31] and another examining the mating competitiveness of radiosterilized males of this species [32], there has been no research regarding the reproductive outcomes of sexual cross-affinity between normal laboratory colonies and wild adults. The present study was performed to examine whether colonization alters the mating ability of *Ae. albopictus* by examining mating compatibility between established laboratory and wild populations. In addition, we also examined the effects of successful mating between these two strains on several fitness traits of both parents and offspring.

## Methods

### Laboratory and wild *Ae. Albopictus*

Two populations of *Ae. albopictus*—a laboratory strain and a wild strain—were used in this study. To establish a new colony, eggs were obtained from the Vector Control and Research Unit (VCRU), University Sains Malaysia. The colony was established 25 years ago from larvae collected from artificial containers in Penang [33,34]. Egg samples oviposited in December 2011 were hatched in dechlorinated water, and newly hatched larvae were raised at a density of 150 per metallic tray (12 cm in diameter and 2 cm in depth). They were fed a diet of powdered mouse pellet and food supplies were performed as described previously [29]. Pupae were sieved and transferred into glass cups lined with moist tissue paper. Adults were placed in cages (30| × |30| × |30 cm) and provided with 10% sucrose solution. Three- to four-day-old females were given blood meals from restrained mice. On day 3 post-blood feeding, plastic cups each lined with an oviposition substrate (cardboard paper sheet), were placed in cages. Eggs were collected, air-dried for 3 days under laboratory conditions (temperature 29°C  ±  3.0°C, relative humidity 75%| ± |1% RH, and photoperiod 13 D: 10 L, 1 h dusk), and stored in plastic desiccation containers. Wild mosquitoes were routinely obtained from fourth instar larvae and pupae collected from outdoor containers in Kampong Teluk Tempoyak (where *Ae. albopictus* accounts for the majority of the mosquito population) and reared to adults in the laboratory. For convenience, we assigned the terms (i) LM (or L♂) and LF (L♀) to males and females reared in the laboratory, and (ii) WM (or L♂) and WF (L♀) to males and females collected from the field.

### Experimental mosquitoes and features

To obtain virgin experimental males and females, both laboratory-reared and wild pupae were placed singly into 1.5-mL Eppendorf tubes containing 0.05 mL of dechlorinated water to ensure sex separation. Pupae were monitored daily, and upon adult emergence, the sex of the mosquitoes was determined. Laboratory-acclimated adult males were pooled in cages labeled LM and emerging females were placed in cages designated as LF. The same procedures were used for adults derived from wild mosquitoes (WM and WF, respectively). Cubic metal wire and mesh cages were used (18| × |18| × |24 cm). Experimental mosquitoes were given access to 10% sucrose solution as described previously [35]. The females were given access to blood meals from immobilized white mice. In all oviposition experiments, glass tubes (2 × 8 cm) lined with a piece of moist cardboard served as oviposition substrates. Tubes were covered with a piece of mesh net at the center of which was an opening filled with a cotton wick covered by a piece of Sealing Film (Parafilm). Females kept in tubes were fed 10% glucose solution from the cotton wick to maintain humidity.

### Parental fecundity

To investigate the fecundity of LF and WF mated with males from their own strain, 26 LF (3 – 4 days old) and 20 LM (2 – 5 days old) were placed in a cage (18 × 18 × 24 cm) and allowed to cohabit. They were given access to a 10% sucrose solution and females were provided with blood meals from an immobilized mouse placed at the bottom center of the cage. Similarly, 21 WF (3 – 5 days old) and 15 WM (2 – 5 days old) were placed in another cage and treated in the same manner. To examine the fecundity of LF when mated with WM, 35 LF (3 – 4 days old) and 29 WM (2 – 5 days old) were allowed to cohabit and feed on sugar and blood as described above. Similarly, 32 WF (3 – 4 days old) and 26 LM (2 – 5 days old) were caged and provided sucrose solution and blood meals as described above.

### Larval eclosion

To inspect egg hatching success, 6 LF (3 – 4 days old) and 6 LM (2 – 5 days old) were placed together in a mosquito cage and given blood meals from a restrained mouse. Other sets consisting of [LF (3 – 5 days old) and WM (2 – 5 days old)], [WF (3 – 5 days old) and WM (2 – 5 days old)], and [WF (3 – 5 days old) and WM (2 – 5 days old)] were placed in three different cages and treated as described above. Six LF (mated with LM), 6 LF (mated with WM), 9 WF (mated with WM), and 10 WF (mated with LM) were placed singly into oviposition containers. After a 24-h oviposition period, females were removed from the tubes and eggs were air-dried for 3 days under laboratory conditions (29°C ± 3.0°C, relative humidity 75% ± 1% RH). Dried eggs were then transferred into 250-mL plastic containers where they were immersed in 20 – 25 mL of hatching medium consisting of 2 mL of 2-day*-*old tap water with 1 – 2 droplets of powdered mouse pellet solution (0.003 g/100 mL).

### Offspring fecundity

To examine offspring fecundity, eggs derived from LF parents in the parental fecundity experiment were hatched in dechlorinated water and first instar larvae were transferred to metallic trays containing 500 mL of water. They were fed 0.15 g of powdered mouse pellets. Upon pupation, the adults were placed in a cage (cage size: 18 × 18 × 24 cm) and given access to 10% sucrose solution. At 3 – 4 days old, females were pooled with WM in a cage at a female to male sex ratio of 10:5. Females were allowed to blood feed for 1 h. On day 3 after blood feeding, oviposition devices were introduced into cages for egg collection.

### Offspring adult lifespan

To determine offspring adult lifespan, newly emerged F1 females from crosses of (i) LF × LM; (ii) LF × WM; (iii) WF × WM, and (iv) WF × LM were allowed to cohabit with males from their own strain [4 individuals of each sex in (i) and 6 individuals of each sex in (ii), (iii), and (iv)] in mosquito cages. They were maintained under laboratory conditions (29°C ± 3.0°C, relative humidity 75% ± 1% RH) and provided 10% sucrose solution.

### Wing length

Wing length of fifteen individuals of both sexes of laboratory-acclimated and field-collected *Ae. albopictus* was measured according to the procedure described previously [36]. The length of each wing, either the left or right, from each dead adult mosquito was measured to the nearest 0.1 mm under a dissecting microscope (Olympus CX41; Olympus, Tokyo, Japan).

### Data collection and analysis

The distance between the apical notch to the axillary margin excluding the wing fringe (expressed in millimeters) was considered as the wing length in accordance with the previous report by Xue and co-workers [36]. In all experiments, the blood meal digestion period was 3 days. After 2 h of cohabitation, the mouse was removed and mosquitoes were left in the cage. Fully blood-fed females were allowed to digest the blood meals. At the end of the 3-day blood digestion period, gravid females were transferred to oviposition containers. At the end of the oviposition period, the cardboard was removed and examined under a dissecting microscope. The number of eggs laid on the cardboard, those deposited on the edges, and those on the bottom of the glass tubes were scored as oviposition responses. The mean values of these numbers were used as measures of fecundity. In Experiment 5, the number of eggs that hatched was determined after 24 h of immersion by counting the number of first instar larvae. These numbers were used to calculate egg hatching rate as the number of hatched eggs divided by the total number of eggs (unhatched + hatched) flooded × 100. The resulting proportions were arcsine transformed before analysis, as described by Dobson and colleagues [37]. In the sixth experiment, dead female(s) were counted on a daily basis until all had died. The adult lifespan was considered as the number of days between adult emergence and death [38].

### Statistical analysis

The differences in fecundity (parents and offspring), egg hatching success, and adult lifespan (males and females) in the different mating pairs and according to body size were investigated by analysis of variance (ANOVA) using the Systat v.11 statistical software package [38,39]. In all analyses, *P* < 0.05 was taken to indicate statistical significance. Where necessary, means ± SE were separated using Tukey’s honestly significant difference (HSD) test.

## Results

### Parental egg production

**Figure 1:**
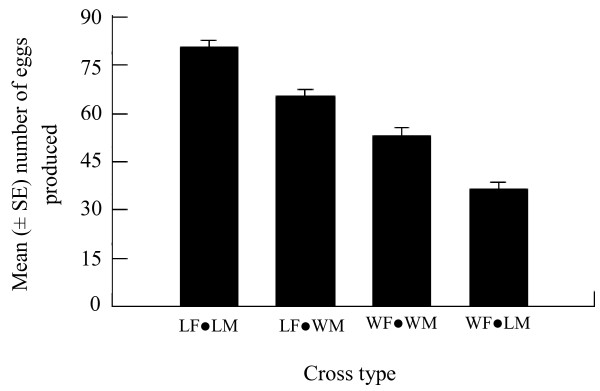
**Numbers of *Ae. albopictus *eggs (mean**| **±** |**SE) produced by laboratory-reared and wild females in interstrain matings, laboratory-reared females mated to wild males (WM), and wild females mated with laboratory-reared males (LM) in interstrain matings.**

Egg production varied significantly with mating pair type (ANOVA, DF = 1, P < 0.001). For the different mating pairs, there was a steady decrease in egg production when progressing from laboratory pairs (80.46 ± 6.82 eggs; range 17–128) to laboratory females (LF) mated with wild males (WM) (65.48 ± 5.44 eggs; range 12–138) to wild pairs (52.90 ± 7.10 eggs; range 9–129) to wild females (WF) mated with laboratory males (LM) (36.50 ± 5.84 eggs, range 0–139). No significant difference in egg production was observed between laboratory pairs and LFs mated with WMs (Tukey HSD, P = 0.304). The mean number of egg produced by the laboratory pairs was significantly greater than those obtained from the wild pairs (Tukey HSD, P = 0.027) and WFs mated with LMs (Tukey HSD, P < 0.001). There was numerically more eggs produced by LFs mated with WMs than the wild pairs, but the difference had no statistical significance (Tukey HSD, P < 0.516). In crosses involving wild mosquitoes, there was a tendency that more eggs were generated by WFs mated with WMs than WFs mated with LMs. However, this difference was insignificant (Tukey HSD, P = 0.296) (Figure 1).

### Offspring egg production

**Figure 2:**
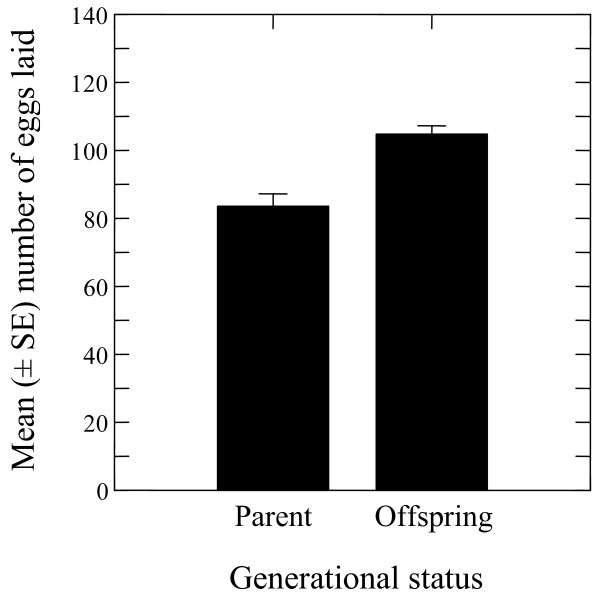
**Numbers of eggs (mean**| **±** |**SE) produced by primary female offspring of laboratory-reared *Ae. albopictus *wild males (LM).**

The mean number of eggs laid by the offspring of LF mated with WM (LF × WM) was 83.57 ± 15.12 eggs; lower and upper CI 46.55 and 120.58, respectively) was lower than the mean egg production of the offspring of WF mated with WM (WF × WM) (104.77 ± 10.21 eggs, lower CI 12.33; upper CI 81.22), but there was no significant difference between the means (Figure 2).

### Egg hatch success

**Figure 3:**
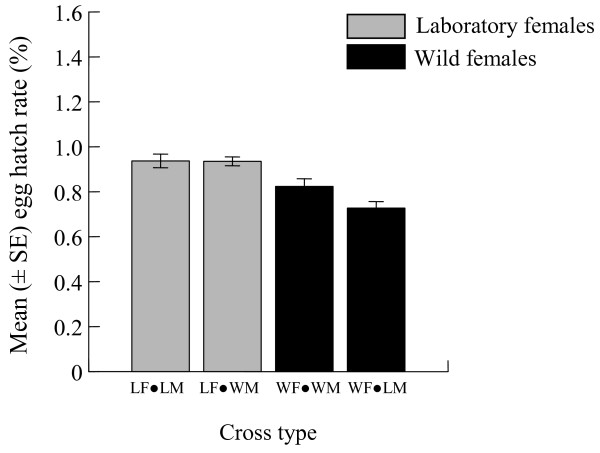
**Mean (± SE) hatch rates of *Ae. albopictus *eggs derived from laboratory-reared intrastrain and interstrain mating pairs.**

The mean hatching rate of eggs derived from LF × LM crosses (0.93% ± 0.08%, range 0.69% – 1.14%) was higher than that of eggs from wild pairs (0.82% ± 0.09%, range 0.47% – 1.18%), but the difference was not significant (ANOVA, DF = 1, *P* = 0.397). Eggs from the laboratory pairs and LF × WM crosses showed similar hatching rates (ANOVA, DF = 1, *P* = 0.985). Egg hatching tended to be more successful among eggs from LF × WM pairs; however, there was no significant difference in larval eclosion rate between eggs from LF × WM and WF × WM crosses (ANOVA, DF = 1, *P* = 0.369). The mean egg hatch fraction from wild pairs was higher than that of eggs from from WF × LM crosses (0.72% ± 0.07%, range 0.34% – 1.01%), but the two means did not differ significantly (ANOVA, DF = 1, *P* = 0.432) (Figure 3).

### Longevity

**Figure 4:**
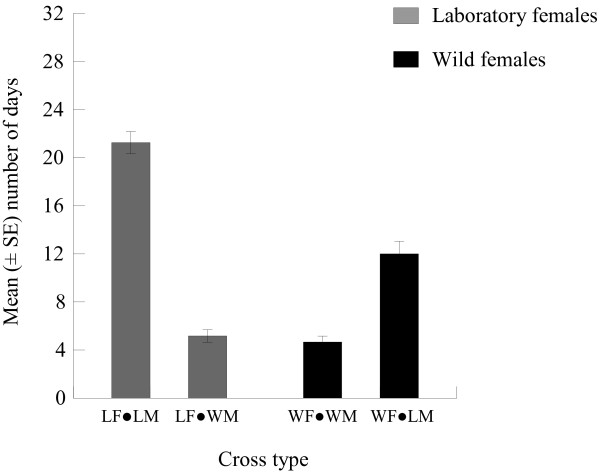
**Adult life spans (mean**| **±** |**SE) of primary female offspring of laboratory-reared *Ae. albopictus *mated with wild adults (WF and WM).**

The adult lifespan of the offspring from laboratory parental pairs (21.25 ± 2.83 days) was markedly longer than that from wild pairs (4.66 ± 1.56 days) (ANOVA, DF = 1, *P* = 0.001). The mean adult life span of female offspring from LF × WM (5.16 ± 1.66 days) was significantly shorter than that of their counterparts derived from laboratory pairs (ANOVA, DF = 1, *P* = 0.001). The mean adult lifespan of female offspring derived from WF × LM crosses (12.00 ± 3.32 days) was longer than that of female offspring derived from wild pairs, but the difference was not significant (ANOVA, DF = 1, *P* = 0.074) (Figure 4).

### Body size

**Figure 5:**
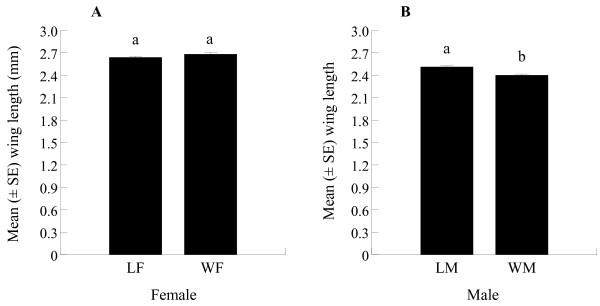
**Wing length (mean ± SE) of laboratory-reared and wild females (A) and males (B) of *Ae. albopictus.*** Bars with the same number are not significantly different (*P* < 0.05) based on Tukey’s test for comparison of means.

The mean wing length of WF (2.68 ± 0.033 mm; range 2.56 – 2.89) was similar to that of LF (2.64 ± 0.017 mm; range 2.54 – 2.71) (ANOVA, DF = 1, *P* = 0.276). LM had a mean wing length of 2.51 ± 0.017 mm, which was significantly greater than that of the laboratory counterparts (2.40 ± 0.021 mm (Figure 5).

## Discussion

The results of the present study indicated that stenogamous laboratory-reared *Ae. albopictus* can successfully mate with their wild counterparts. Females from the laboratory-raised colony laid eggs in considerable numbers when mated with wild males. Mating between WF and LM yielded large numbers of eggs. The female offspring that resulted from these cross-mating events showed increased fecundity, particularly those derived from the mating of WF  ×  WM and mated with WF.

LF produced more eggs than their wild counterparts when both mated with WM. Laboratory-maintained insect strains are thought to have greater energy stores than wild strains as they are exposed to less severe environmental conditions. In the laboratory environment, the high-nutrient larval conditions result in the production of large-sized individuals. Fecundity has often been correlated with body size in mosquitoes. In general, small females produce fewer eggs and have delayed ovarian development [40,41], and larger females produce more eggs over a lifetime than small individuals [42]. In this study, body size measurements indicated that females derived from wild pupae (WF) and those in the laboratory (LF) were of similar size, strongly suggesting that female body size may not have played a role in the observed differences in egg production between wild and laboratory strains.

Crosses between WF × LM and WF × WM showed comparable fecundity. In addition, the fecundity of wild pairs was similar to that of primary offspring of LF mated with WM, similar to the observations reported previously [19]. These researchers investigated the fitness and sexual cross-compatibility between wild and laboratory populations of the malaria vector, *Anopheles arabiensis* that originated from material collected in 1994, and noted that the reproductive fitness of the laboratory strain was not significantly modified with respect to the wild pairs. They attributed the decreased variations apparent in the fitness of the laboratory strain to the reduction in genetic variation generally inherent in laboratory colonies. In addition, Muhenga and colleagues [19] observed increased insemination rates of wild females by laboratory-acclimated males, and suggested that this increased mating success was a result of a high degree of genetic compatibility between the two strains. It is possible that there was a similar genetic affinity between the laboratory and wild populations of *Ae. albopictus* used in this study. It is interesting to note that the colony used was far older than that in the study of Muhenga and co-workers [19]—both LF and LM were derived from a < 25-year-old laboratory colony and WF were collected in the field as pupae in early 2012. Mating between the laboratory pairs and LM with WF was highly productive. The hatching success rate of eggs from WF mated with LM was similar to that of eggs from WF mated with WM. In addition, eggs derived from LF × WM and LF × LM crosses had similar hatching success rates; there were no significant differences in egg production between primary female offspring and parents when both were mated with WM. Finally, the adult lifespan of female offspring of WF × WM crosses was similar to that of their counterparts from WF × LM crosses. Taken together, these results suggest that *Ae. albopictus* has retained both reproductive and physiological fitness while being kept in the laboratory for 25 years.

Similar to *Ae. albopictus*, many arthropods known to be involved in the transmission of pathogens to humans, domestic animals, and wildlife are maintained in laboratories for research purposes [13]. Containment is necessary to gain information regarding their behavior, life cycle, infectivity, and susceptibility to infection-blocking strategies [15]. Previous research in *Ae. albopictus* has helped us gain a better understanding of the medical importance of this species. This mosquito is known to transmit at least 22 human arboviruses, including flaviviruses (dengue virus, yellow fever virus, Japanese encephalitis virus, and West Nile virus) and togaviruses (Ross River virus) [43]. *Ae. albopictus* is also a vector of alphaviruses, such as Chikungunya [44] and equine fever [45]. No vaccines or preventative drug treatments are currently available for most of these arboviral infections. This mosquito species is also highly invasive [46,47]. Although it is indigenous to Southeast Asia, *Ae. albopictus* has traversed the world over the past 30 years [46] and is listed by the World Conservation Union as one of the world’s most invasive species [48]. Ecological studies have indicated that climate does not significantly constrain the establishment of this mosquito [47,49], which is capable of overwintering in cold climates [50,51]. Another specific characteristic of this mosquito species is that it is capable of transovarial transmission of dengue serotypes 1, 2, 3, and 4 to its offspring [52]. Thus, an infected female can transmit the virus to the next generation via its eggs. In addition, male-to-female sexual transfer has also been documented in this mosquito; males experimentally infected with all dengue serotypes transmitted their infection to females through mating [53]. In this study, LFs were more fecund than WF when both mated with WM. Clearly, in nature, increased fecundity will tend to result in higher cumulative offspring rates, and the mosquito populations are more likely to persist in nature with increased egg production*.* Epidemiologically, if LF is infected with dengue virus, the increased egg production observed when LF or their offspring mate with WF will, therefore, lead to high population densities, but also propagation and maintenance of virus infection.

Pathogen-infected vectors represent an immediate threat, but even uninfected arthropods that escape captivity can establish populations that subsequently transmit pathogens [15]. Despite guidelines for safe containment of colonies to prevent inadvertent escape [14,15], there have been a few instances where an insect has escaped from a laboratory and resulted in a significant public health issue. One of the most striking examples is *Rhodnius prolixus*; indigenous to northern South America, this Chagas disease vector was introduced into Central America by escape from a laboratory in El Salvador in 1915 [13]. Work with colonies of *Aedes* mosquitoes is associated with a high risk of the escape of eggs, which measure only about 1 mm, from the laboratory. In the case of *Aedes* dengue vectors, it is known that eggs can survive in the environment for several months and then hatch at the onset of rain [16]. Therefore, the World Health Organization has suggested that eggs be taken into consideration in arthropod containment measures [13].

## Conclusions

In addition to providing insights into the reproductive biology of *Ae. albopictus*, the present study underlined the importance of revisiting the issue of insect containment, particularly with regard to preventing escape, as this can result in significant public health issues. The present study indicated that *Ae. albopictus* can retain its reproductive and physiological fitness for 25 years in the laboratory environment, that eggs derived from interstrain mating events of LF and LM with wild adults show increased hatching success rates, and that female offspring of WF × WM crosses have long lives. These observations in combination with the extreme sexual aggressiveness of the males, illustrate the potential of *Ae. albopictus* as a suitable candidate for SIT application. *Ae. albopictus* has been reported to successfully mate with heterospecific females. In mixed populations, among 78 mating *Aedes polynesiensis* females, 56% involved *Ae. albopictus* males [54]. There was a mating rate of 90% of *Ae. polynesiensis* by *Ae. albopictus* males in the presence of their own females. In a related study, dissection of the spermathecae indicated heterospecific insemination between *Ae. albopictus* and *Aedes aegypti* with production of eggs [55]. Interspecific mating between *Ae. albopictus* males and *Ae. aegypti* female was detected in the field using the mark-release-recapture technique. 3 days after the release of virgin *Ae. aegypti* females into a field site containing only *Ae. albopictus*, 100% of the captured females were inseminated [56,57]. Sexual aggressiveness would lead to sperm depletion for further inseminations in the presence of refractory females. If a female fails to give the proper cues indicating receptivity due to the presence of heterospecific sperm, conspecific mating and fecundity may be reduced. Considerably high mating rates of *Ae. polynesiensis* and of *Ae. aegypti* by *Ae. albopictus* males [55,57] have been reported. These hybrid matings in some cases resulted in egg production. In failed mating cases, examination of spermathecae of *Ae. aegypti* females inseminated by *Ae. albopictus* males revealed the presence of dead sperm. This dead sperm could function as a mating plug, known to function as a visual deterrent to males [58] and to prevent re -mating by females [59]. The release of non-sterilized *Ae. albopictus* males (such as those based on SIT) may be a practical strategy for the control of *Ae. aegypti* populations, and has several important implications, i.e., the use of such males in place of sterilized males has the potential to not affect competitiveness in contrast to irradiation [60], and may reduce insecticide use, thereby reducing costs related to irradiator use.

## Competing interests

The authors declare no competing financial interests.

## Authors’ contributions

Designed the experiments: HD NBR AHA CSMR RMV SA. Carried out the experiments: HD NBR WFZ SR ARS HA. Analyzed the data: HD TS FM. Carryied out re-analysis and answers to reviewers: HD IAG SR NFNR. All authors read and approved the final version of the manuscript.
